# Hippo signaling pathway is altered in Duchenne muscular dystrophy

**DOI:** 10.1371/journal.pone.0205514

**Published:** 2018-10-10

**Authors:** Gian Luca Vita, Francesca Polito, Rosaria Oteri, Roberto Arrigo, Anna Maria Ciranni, Olimpia Musumeci, Sonia Messina, Carmelo Rodolico, Rosa Maria Di Giorgio, Giuseppe Vita, M’Hammed Aguennouz

**Affiliations:** 1 Nemo Sud Clinical Centre for Neuromuscular Disorders, Messina, Italy; 2 Unit of Neurology and Neuromuscular Diseases, Department of Clinical and Experimental Medicine, University of Messina, Messina, Italy; University of Louisville School of Medicine, UNITED STATES

## Abstract

Hippo signaling pathway is considered a key regulator of tissue homeostasis, cell proliferation, apoptosis and it is involved in cancer development. In skeletal muscle, YAP, a downstream target of the Hippo pathway, is an important player in myoblast proliferation, atrophy/hypertrophy regulation, and in mechano-trasduction, transferring mechanical signals into transcriptional responses. We studied components of Hippo pathway in muscle specimens from patients with Duchenne muscular dystrophy (DMD), Becker muscular dystrophy, limb-girdle muscular dystrophy type 2A and type 2B and healthy subjects. Only DMD muscles had decreased YAP1 protein expression, increased LATS1/2 kinase activity, low Survivin mRNA expression and high miR-21 expression. In light of our novel results, a schematic model is postulated: low levels of YOD1 caused by increased inhibition by miR-21 lead to an increase of LATS1/2 activity which in turn augments phosphorylation of YAP. Reduced amount of active YAP, which is also a target of increased miR-21, causes decreased nuclear expression of YAP-mediated target genes. Since it is known that YAP has beneficial roles in promoting tissue repair and regeneration after injury so that its activation may be therapeutically useful, our results suggest that some components of Hippo pathway could become novel therapeutic targets for DMD treatment.

## Introduction

The Hippo signaling pathway is considered a key regulator of tissue homeostasis, cell proliferation and apoptosis, and its alterations participate to cancer development. Yes-associated protein 1 (YAP or YAP1) is a downstream target of the Hippo pathway and acts as a transcription co-activator [1]. YAP can be down-regulated through phosphorylation by the large tumor suppressor 1/2 (LATS1/2) kinase [2]. Phosphorylated YAP interacts with cytoskeletal proteins and is maintained in the cytoplasm. Non-phosphorylated YAP translocates to the nucleus where it exerts its regulatory function on many transcription factors such as TEAD family, being TEAD and YAP transcriptional coactivators in most of genomic loci [3]. Important target genes of YAP are Cyclin D1, Birc5, and myogenic transcription factor Myf5 [4].

In skeletal muscle from different animal models, YAP appeared to be a prominent player in mechano-transduction, transferring mechanical signals into transcriptional responses. Moreover, YAP is involved in muscle development and regeneration, and regulates activation, proliferation and differentiation of satellite cells [4]. Hippo signaling is similarly crucial in mature skeletal muscle homeostasis: its misregulation can cause atrophy or hypertrophy. Mammalian sterile 20-like kinase 1 (MST1), a focal member of Hippo pathway, participates to the development of atrophic changes in denervated muscle, as result of the activation of Forkhead box O3 (FOXO3) transcription factors [5].

Very recently, it has been proposed that modulation of the Hippo pathway effectors YAP and transcriptional activator with PDZ binding motif (TAZ) may, in part, provide a mechanistic explanation for the hypertrophic effects of resistance exercise through changes in the rates of muscle protein synthesis and satellite cell activity [6]. Resistance exercise affects metabolic, hormonal and mechanical responsive elements, all mediators of YAP and TAZ activity in epithelial cells [7]. Some, or all, of these inputs also alter YAP and TAZ activity in skeletal muscle during resistance exercise. Manipulation of the metabolic, hormonal or mechanical pathways engaged might provide insight into the mechanisms regulating YAP and TAZ activity in skeletal muscle that could be exploited for therapeutic benefit in isolation, or combination, with exercise-based interventions [6].

During muscle differentiation, YAP phosphorylation is augmented, which is important for myoblast differentiation [8]. YAP expression increases when satellite cells are activated but declines when differentiation is starting and therefore expression of YAP stimulates proliferation but prevents differentiation [9]. In contrast, YAP knockdown strongly decreases myoblasts proliferation [9]. A microarray study suggested that transcription of many genes upstream to YAP are amplified in *mdx* muscle, the murine model of Duchenne muscular dystrophy (DMD), and it has been postulated that dystrophic muscle with increase of inflammatory and regenerated/degenerated cells activates the Hippo pathway [10]. The phosphorylation of YAP increases after myostatin and activin blocking and also in exercised muscle, and *mdx* mice display increased content of phosphorylated and especially total amount of YAP protein [10]. These results suggest that Hippo signaling may have an important but yet uncertain regulatory role in dystrophic skeletal muscle.

So far the literature does not report any data about YAP expression in DMD and other muscular dystrophies. The goal of the present study was to test the hypothesis that altered YAP signaling may contribute to dystrophic pathogenesis in DMD muscle, becoming a pharmacological target of dystrophinopaties.

## Materials and methods

### Study subjects

We studied vastus lateralis muscle samples, stored at −80° C, from 5 patients with DMD (age range: 4–6 years), 5 patients with Becker muscular dystrophy (BMD) (age range: 5–11 years), 5 patients with limb-girdle muscular dystrophy type 2A (LGMD2A) (age range: 14–55 years), and 5 patients with limb-girdle muscular dystrophy type 2B (LGMD2B) (age range: 26–50 years). DMD, BMD, LGMD2A and LGMD2B had been diagnosed on clinical features, muscle biopsy including immunocytochemistry and Western blot, and genetic analysis. 5 muscle samples taken from healthy subjects (age range: 3–50 years), without muscle disorder and undergoing orthopaedic surgery, were used as controls. All adult individuals and the parents of all participants under age 18 had provided written, informed consent for the use of their muscle samples in research. The review board of the Department of Neurosciences, University of Messina, reviewed and approved the study.

### Western blotting

About 30 mg of muscle specimen were homogenized in a glass tube with Teflon dounce pestle in 20 volumes of a detergent saline buffer containing lysis buffer (20 mM KCl, 15% SDS and 5% β-mercaptoethanol). Samples were heated 10 min at 100°C and then centrifuged for 10 min at 12,000 g. Protein concentration of tissue homogenate was determined by Lowry assay. Protein balanced samples were prepared for sodium dodecyl sulfate polyacrylamide gel electrophoresis (SDS-page) in two-fold loading buffer containing 0.25 M Tris (pH 6.8), 0.2 M DTT, 10% SDS, 0.02% bromophenol blue, and 20% glycerol in distilled water. Fifty micrograms of proteins per line were routinely resolved by 12% SDS-PAGE for 2 h at 130 V.

Following electrophoresis, separated proteins were laterally transferred to nitrocellulose membranes in transfer buffer containing 0.192 M glycin and 0.025 M Tris at pH 8 with 20% methanol. At a voltage of 100 V for 1 h at 4°C, blots were blocked for 1 h at room temperature (RT) in a saturating solution containing 0.9% NaCl, 1% bovine albumin serum and 0.05% Tween-20. Membranes were then incubated with monoclonal antibodies against YAP1 (1:400) (TermoFisher Scientific, Waltham, MA, USA), Survivin (1:2,000) (Santa Cruz Biotechnology, Inc., Santa Cruz, CA, USA), and human Actin (1:20,000) (TermoFisher Scientific) at 4°C overnight. Blots were then washed and the second incubation was performed in blocking buffer containing, respectively, 1:20,000, 1:10,000 and 1:10,000 dilution of the appropriate HRP-conjugated secondary antibody (Sigma-Aldrich, Missouri, USA) at RT for 1h. Membrane were developed using ECL Plus Western Blotting Detection kit (Amersham Biosciences, Little Chalfont, UK) following the manufacturer's protocol. Quantification of the detected protein was carried out using the Alpha Digi Doc apparatus (Alpha Innotech Corp, San Leandro, CA, USA) for image acquisition (8 bit gray-scale), and by the ImageJ software for densitometric analysis. The results were finally expressed as relative density for each sample after β-actin normalization.

### LATS1/2 kinase activity assay

To assess LATS1/2 enzyme activity, immunoprecipitation followed by western blotting of phosphorylated YAP1 was performed. Muscle specimens were lysed in 1% Nonidet P-40 lysis buffer supplement containing 1 mM DTT and 1× phosphatase inhibitor (Sigma-Aldrich). For immunoprecipitation-kinase assay, 100 μg of protein lysate were mixed with 2 μg of monoclonal antibody against YAP1 (1:400) (Santa Cruz Biotechnology, Inc.) together with Protein G beads and incubated at 4°C for 3 h. Then, beads were washed twice with 1% Nonidet P-40 lysis buffer, 1 mM DTT; once for 10 min with 1% Nonidet P-40 lysis buffer, 500 mM NaCl, 1 mM DTT at 4°C; and twice with 20 mM Tris-HCl (pH 7.4), 1 mM DTT, 1× phosphatase inhibitor. The washed beads were mixed with 2 μg of YAP-GST substrates in a kinase buffer (20 mM Tris-HCl (pH 7.5), 5 mM MgCl2, 5 mM MnCl2, 1× phosphatase inhibitor, 2 mM DTT, 10 μM ATP, 5 μCi of [γ-32P]ATP) and incubated at 30° C for 30 min. The reaction was stopped by adding 7 μl of 5× SDS sample dye, boiled at 100°C for 5 min, and subjected to SDS-PAGE. After electrophoresis, the proteins were transferred to a nitrocellulose membrane, and exposed to autoradiograph film for 0.5–2 h. to test phosphorylation of YAP1 by LATS1/2.

### Animal studies

Quadriceps, biceps, diaphragm, gastrocnemious and extensor digitorum longus (EDL) muscles previously frozen in liquid nitrogen-cooled isopentane and stored at -80°C were used for YAP1 western blotting and LATS1/2 kinase activity assay (see procedures above). Muscle specimens had been removed from 10-15-week old male *mdx* (n. 4) and wild-type C57BJ/10ScSn (WT) (n. 4) mice from The Jackson Laboratory (Bar Harbor, Maine, USA) as part of our previous experiments [11]. Membranes were incubated with monoclonal antibodies against YAP1 (1:500) (Santa Cruz Biotechnology, Inc.) and mouse Actin (1:5,000) (TermoFisher Scientific) at 4°C overnight.

### Semi-quantitative evaluation

Semi-quantitative evaluation of protein levels detected by immunoblotting was implemented with computer-assisted densitometry (UN-SCAN-IT gel version 6.1; Silk Scientific, Inc., Orem, UT, USA). Data were then acquired and integrated density values expressed as a percentage of densitometric levels using arbitrary densitometric units.

### RNA, cDNA synthesis and microRNA (miR) isolation

Total RNA was extracted from muscle specimens using TRIzol reagent (Invitrogen; Thermo Fisher Scientific, Inc., Waltham, MA. USA), according to the manufacturer's instructions. Entire RNA concentration and integrity were checked using an Agilent Bioanalyzer (Agilent Technologies, Inc., Santa Clara, CA, USA). Successively, 300 ng of total mRNA per sample was reverse transcribed into cDNA using the High Capacity cDNA Reverse Transcription kit (Applied Biosystems; Thermo Fisher Scientific, Inc.). miRNAs were extracted from muscle using the miRVana Isolation kit (Ambion; Thermo Fisher Scientific, Inc.), following the manufacturer's protocol. The enriched miRNAs fraction was converted in cDNA using the TaqMan MicroRNA Reverse Transcriptase kit (Life Technologies; Thermo Fisher Scientific, Inc.).

### Real Time-quantitative polymerase chain reaction (RT-qPCR) of Survivin and miR-21

To validate downstream YAP pathway, Survivin (Birc5) which is one of the prominent target genes of YAP [3,12], and miR-21 which is involved in epigenetic regulation of YAP [13] were studied. RT-qPCR for Survivin Assay ID Hs01125524_m1 was performed using a standard TaqMan PCR kit procedure on an AB-7300 RT-PCR system (Thermo Fisher Scientific, Inc.). Relative fold expression and changes were calculated using 2−ΔΔCtmethod. The expression levels of Survivin were normalized to β-actin housekeeping gene and indicated as fold expression (<0.3 downregulation and >3 upregulation) compared to control normal muscle. Results were represented as Log10 relative quantitative (RQ).

For miRNA quantification, 2 μl of cDNA were used for each specific miRNA TaqMan assay (hsa-miR-21) according to the manufacturer's instructions. All reactions were performed in triplicate. RNU6 small nuclear RNA was used to normalize miRNA expression levels due to its claimed expression stability and its wide use as loading control in published studies [14–16].

### Target prediction tools

miR-21 that target YAP1 was identified by examining the YAP 3′-untranslated region (UTR) with bioinformatics algorithms predicting miRNA target sites [17]. Specifically, four online databases, miRDB (http://mirdb.org/miRDB/), TargetScanHuman (www.targetscan.org), microRNA.org (www.microrna.org) and PicTar (http://pictar.mdc-berlin.de), were used.

### Statistical data analysis

Statistical analysis was performed by GraphPad Prism, version 7.00 (GraphPad Software, La Jolla, CA, USA). Results are expressed as mean ± standard deviation (SD). Statistical multiple comparison between groups was performed by Kruskal-Wallis ANOVA test followed by Dunn’s post hoc test. The relationship between variables was studied using Spearman correlation test. Comparison between groups was performed by Mann-Whitney test for unpaired non-parametric data. A level of significance of p < 0.05 was considered.

## Results

**Fig 1:**
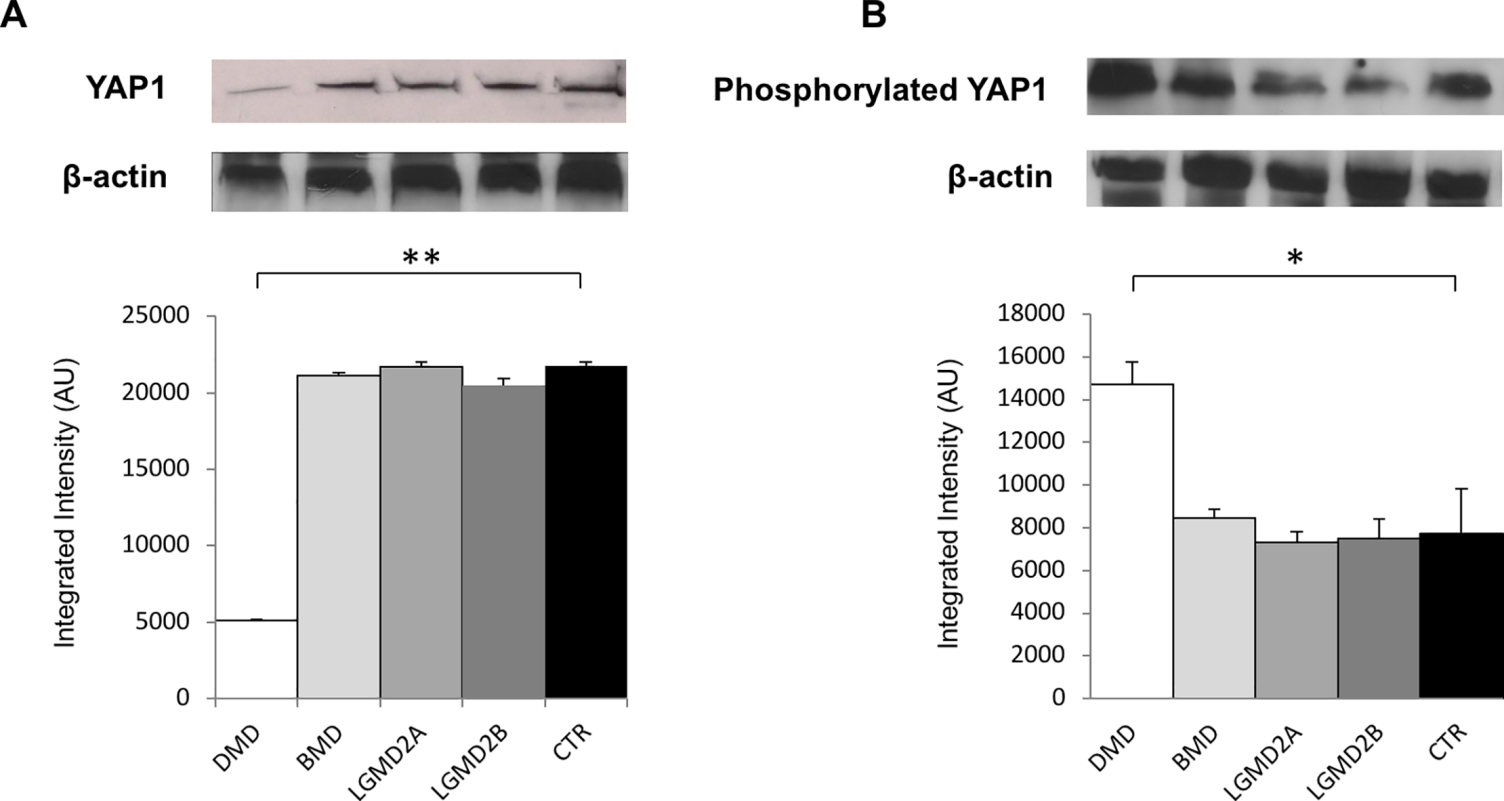
Western blot analysis of YAP1 (A) and LATS1/2 kinase activity assay performed by immunoprecipitation followed by western blotting of phosphorylated YAP1 (B) in human muscular dystrophies (DMD, BMD, LGMD2A, LGMD2B) and normal controls (CTR). Lower panel shows graphs with quantitative data; upper panel shows representative autoradiograms. *p < 0.04; **p < 0.001.

Western blot analysis revealed a 4-fold decrease of non-phosphorylated YAP1 expression in muscle specimens from DMD patients (p < 0.001 vs control normal muscles). No difference was found in BMD, LGMD2A and LGMD2B patients vs. control (Fig 1A).

LATS1/2 kinase activity, assessed by immunoprecipitation followed by western blotting of phosphorylated YAP1, resulted 2-fold increased in DMD patients (p < 0.04 vs control normal muscles). No difference was found in BMD, LGMD2A and LGMD2B patients vs. control (Fig 1B).

**Fig 2:**
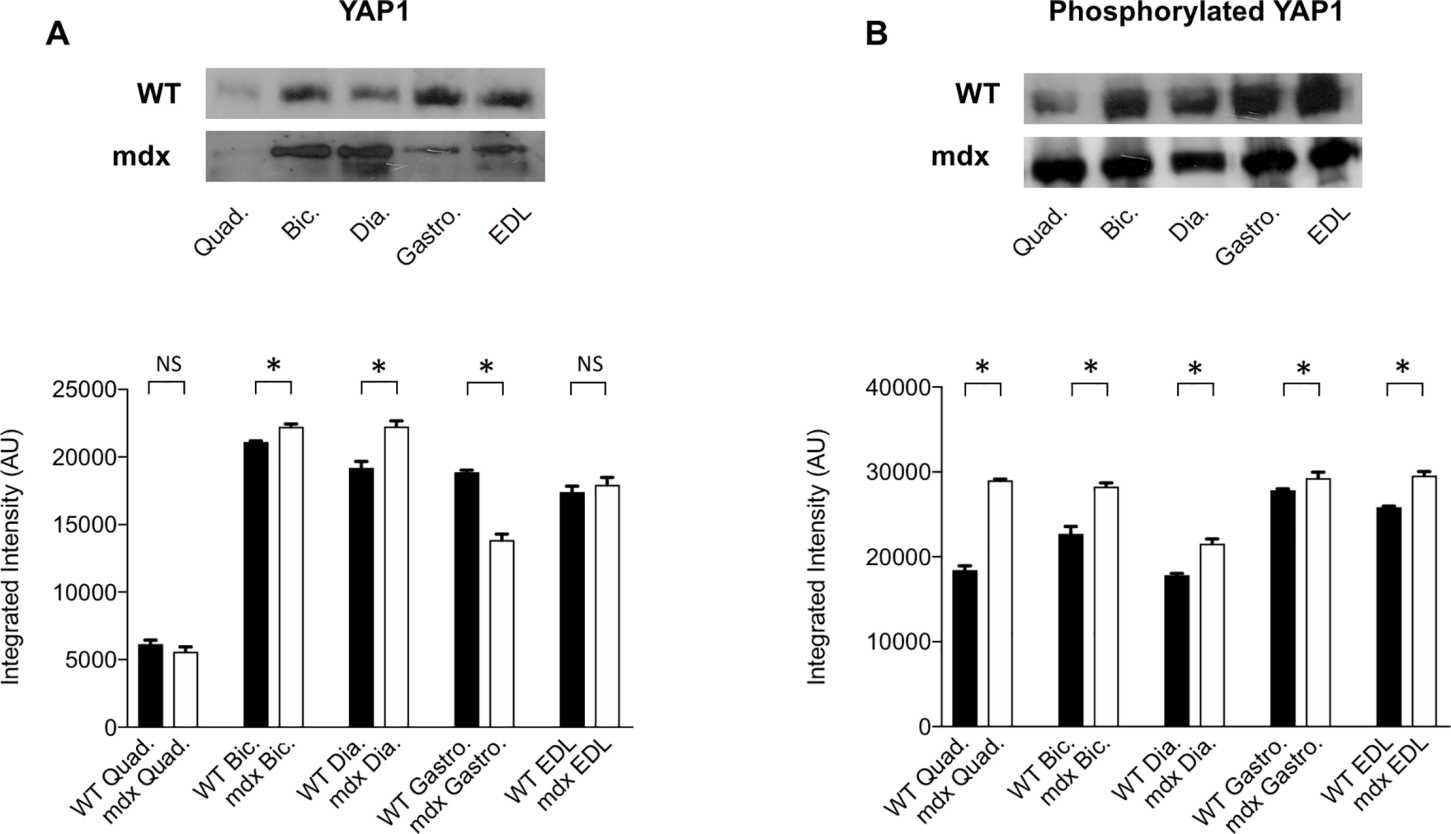
Western blot analysis of YAP1 (A) and LATS1/2 kinase activity assay performed by immunoprecipitation followed by western blotting of phosphorylated YAP1 (B) in quadriceps (Quad.), biceps (Bic.), diaphragm (Dia.), gastrocnemious (Gastro.) and EDL muscles of WT and *mdx* mice. Lower panel shows graphs with quantitative data; upper panel shows representative autoradiograms. *p < 0.03.

Experiments in *mdx* mice showed no significant change of YAP1 expression in quadriceps and EDL muscles compared to WT animals, a 26% decrease in gastrocnemious and 5% and 16% increase in biceps and diaphragm muscles respectively (all p < 0.03) (Fig 2A). LATS1/2 kinase activity, assessed by western blotting of phosphorylated YAP1, resulted significantly increased (p < 0.03) in all five muscles vs. WT mice, with variable degree from 5% in gastrocnemious to 57% in quadriceps (Fig 2B).

**Fig 3:**
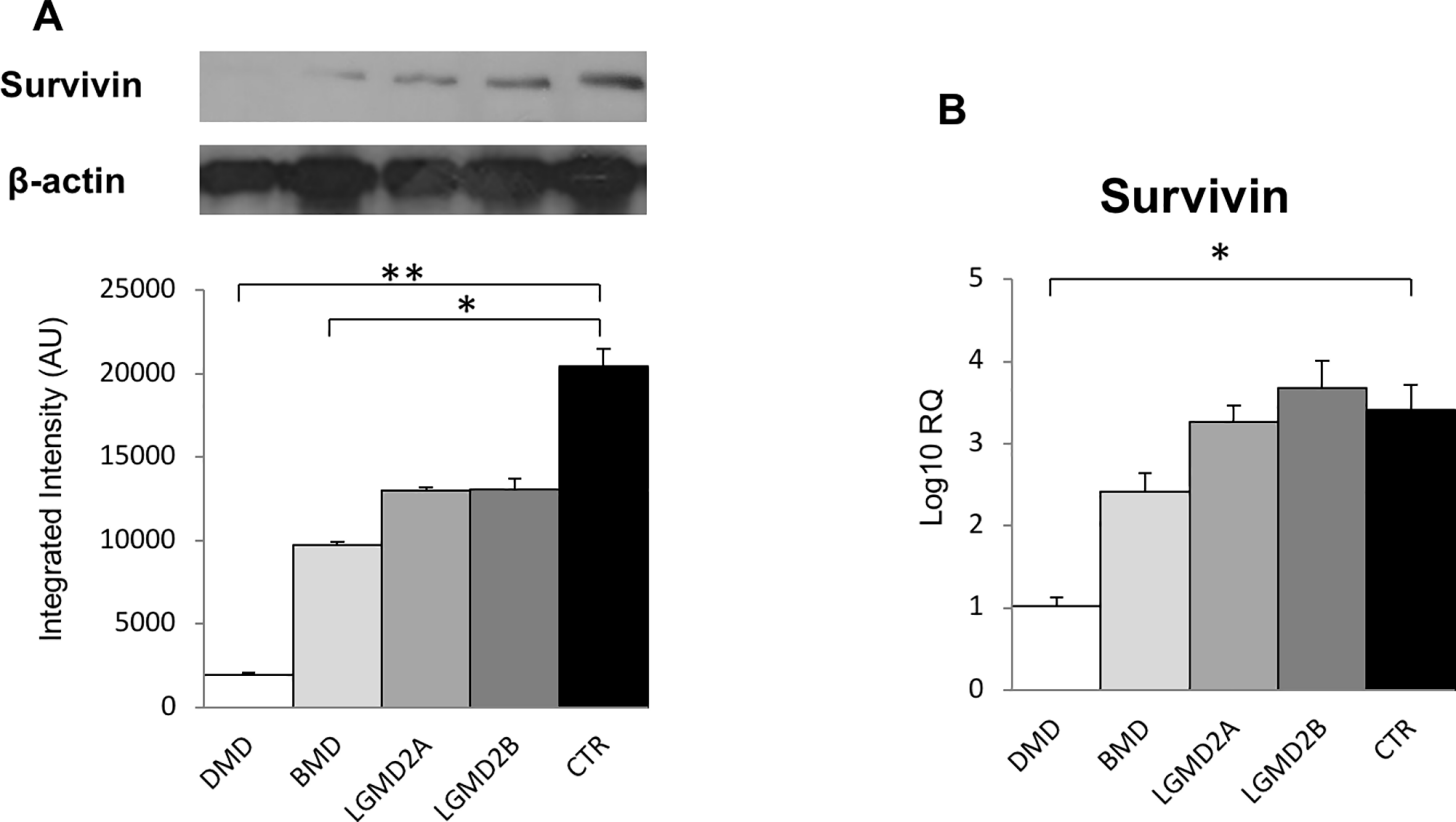
Survivin protein (A) and mRNA (B) levels in human muscular dystrophies (DMD, BMD, LGMD2A, LGMD2B) and normal controls (CTR). *p < 0.01; **p < 0.0001.

**Fig 4:**
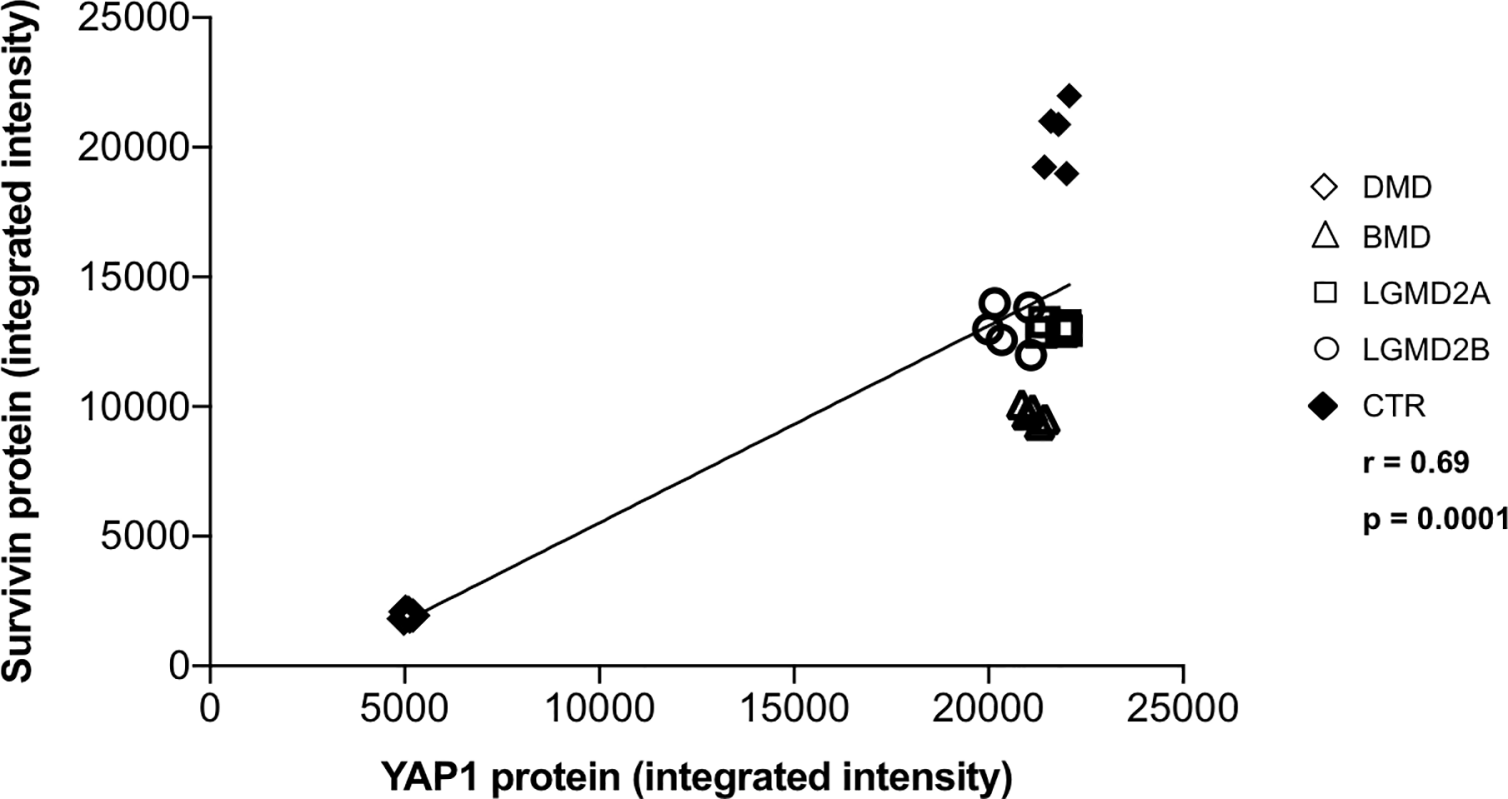
Correlation analysis between Survivin protein and YAP1 protein levels in human muscular dystrophies (DMD, BMD, LGMD2A, LGMD2B) and normal controls. r: 0.69; p < 0.0001.

Since transcription of Survivin is regulated by YAP [3,12,18], its expression was studied. Survivin protein was decreased in DMD (p < 0.0001) and at lower amount also in BMD (p < 0.01) vs control muscles (Fig 3A). RT-qPCR revealed that Survivin mRNA level was 3-fold lower in the muscles from DMD patients (p < 0.0001 vs control muscles) (Fig 3B). Fig 4 shows the significant correlation between Survivin protein and YAP1 protein levels in all twenty-five muscle specimens from patients and normal controls (r: 0.69; p < 0.0001).

**Fig 5:**
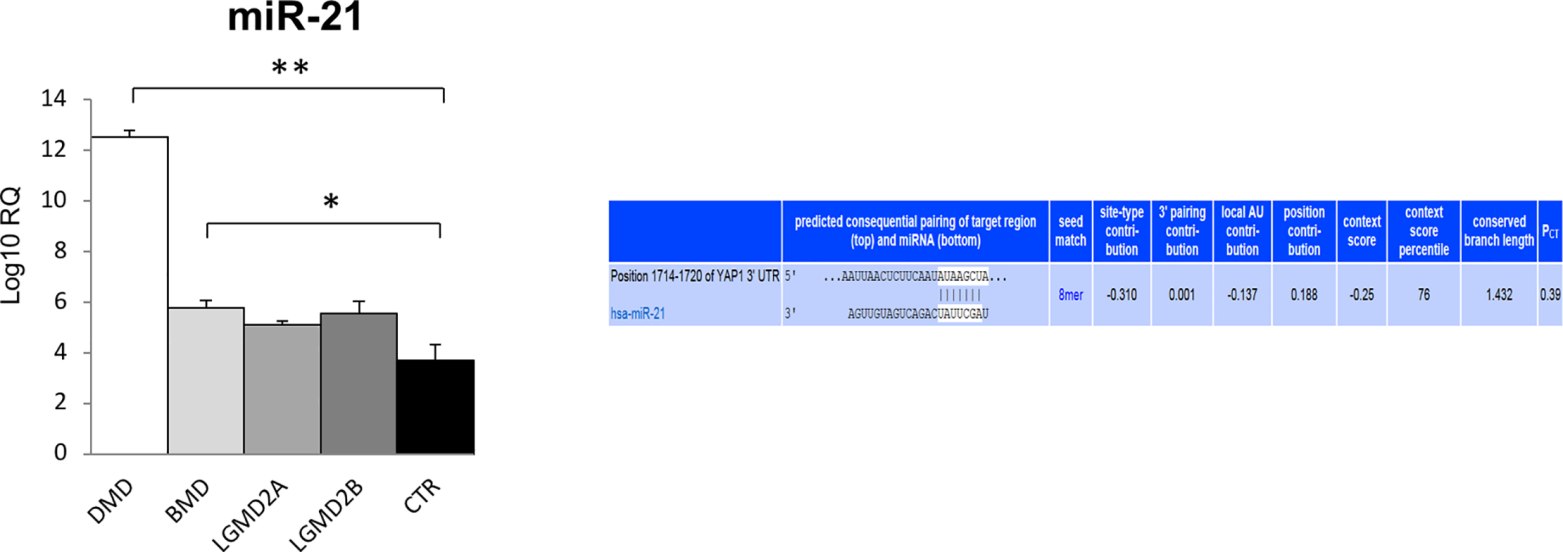
miR-21 expression in human muscular dystrophies (DMD, BMD, LGMD2A, LGMD2B) and normal controls (CTR). Right panel: Aligment details with 3′-UTR region of YAP1. YAP1 is direct target of miR-21, according to computational target prediction (www.targetscan.org). *p < 0.03; **p < 0.0001.

miR-21 is involved in epigenetic regulation of YAP1 at two levels: i) miR-21 suppression results in elevated levels of LATS1/2, which down-regulates YAP through phosphorylation [13]; ii) computational target prediction identifies homology between miR-21 and 3'-UTR of human YAP1 mRNA (www.targetscan.org). RT-qPCR revealed that miR-21 expression was significantly more than 3-fold increased in the muscles from DMD patients (p < 0.0001 vs control muscles). A lower increase was found in BMD patients vs. controls (p < 0.03) (Fig 5).

No correlation has been found, within the single disease type, between severity, duration and progressiveness of myopathy and altered Hippo pathway activation.

## Discussion

The best studied role of YAP in skeletal muscle is as a regulator of myoblast proliferation and terminal differentiation. Several studies together demonstrate that YAP and TAZ activity are increased as satellite cells/myoblasts proliferate and that further increasing their activity, by over-expression of mutant YAP/TAZ proteins that cannot be inhibited by LATS1/2, results in an enhanced rate of myoblast proliferation [8,9,19]. Moreover, Hippo signaling pathway is similarly crucial in mature skeletal muscle homeostasis, its misregulation causing atrophy or hypertrophy [5], and in mechano-transduction, transferring mechanical signals into transcriptional responses [4].

Very little is known about YAP expression in human skeletal muscle. A recent proteomics study using single muscle fibres has reported that YAP protein expression in slow-twitch muscle fibres is ~ 2-fold higher than in fast-twitch 2A fibres from young subjects [20]. In the same study, it was also shown that YAP is ~50% lower in both these muscle fibre types in aged subjects compared to younger controls. Possible fibre type-dependent differences in the regulation of YAP, and a potential role played by reduced YAP in the age-dependent loss of skeletal muscle mass (i.e. sarcopenia) have been suggested.

Levels of total YAP and phosphorylated YAP are elevated in the *mdx* mouse model of DMD, supporting a role for YAP in the setting of skeletal muscle degeneration/regeneration [10]. These findings are of particular interest given recent studies linking Hippo signalling to Agrin, an essential element regulating stability and organisation of the neuromuscular junction and the dystroglycoprotein complex (DGC) in cardiac tissue [21–23]. DMD, a progressive muscle wasting disease, is due to the absence of dystrophin protein, leading to recurrent muscle fibre damage during contraction and to loss of ambulation by the 13th year and to death, usually in early adulthood [24]. Although the primary genetic defect is known, the cellular and molecular mechanisms which characterize the disease are not completely understood and a successful treatment remains to be developed. Pathological hallmarks of the dystrophic process include necrosis, phagocytosis, inflammation and initial efficient regeneration followed by exhaustion, and connective and adipose tissues replacement [25]. The inflammatory response to fibre damage in DMD is an engaging candidate mechanism for disease worsening and different steps of inflammatory cascade, such as B-4, COX, LOX, MAPK, TNF-α, reactive oxygen species, and nuclear factor-κB signaling factors, are considered possible therapeutic targets to be joined with exon skipping therapy or protein restoration therapy [11, 26–28].

We demonstrated for the first time that YAP1 expression is decreased and LATS1/2 kinase activity increased in DMD muscles but not in muscles from patients with other types of muscular dystrophy, stressing the specificity of the results. Besides, DMD results were fairly confirmed in *mdx* mice with increased LATS1/2 kinase activity in five different muscles and variable results of YAP1 expression in them, from a decrease to no difference and to a mild increase when compared to WT mice. Our *mdx* results resulted also in agreement with previous report [10]. In mammals, LATS1/2 binds and phosphorylates YAP1 affecting its transcription regulation. As a result, phosphorylated YAP1 is retained in the cytoplasm, undergoing proteasomal degradation. Different stimuli may inhibit LATS1/2-mediated YAP1 phosphorylation and permit YAP1 to enter the nucleus and activate the transcriptional programs involved in cell survival and proliferation [29]. Moreover, YAP activity may be also modulated by other regulators such as deubiquitinase YOD1. YOD1 stabilizes ITCH, facilitating ITCH-mediated LATS1/2 ubiquitination and degradation, and as result YAP level increases [30].

Consistent with decreased YAP1 expression and increased LATS1/2 kinase activity, we found Survivin protein and mRNA under-expressed and mir-21 over-expressed in DMD muscles in a specific manner. In accordance with these results, a lower decrease of Survivin protein and a lower increase of mir-21 expression were found in BMD, which is the allelic milder form of the same disease [24,25]. Survivin is able to promote cell cycle and inhibit apoptotic caspase functions, and is mainly found in developing tissue and cancer [31–32]. Since YAP and Survivin increase in a parallel way in liver tumors [18], it is interesting to ascertain if YAP complements with Survivin in other diseases. We found a strong correlation between Survivin protein and YAP1 protein levels also in normal and diseased skeletal muscles.

miRs are non-coding, small RNAs, able to destroy mRNA of target genes through binding to a specific region, so regulating the expression and function of downstream genes. miRs contribute to the control of development, cell apoptosis, proliferation, differentiation and other essential cell activities [33,34]. Recently, it was discovered that many miRs play a crucial role in the development of cancer and other diseases [35]. The YOD1- ITCH-LATS1/2-YAP/TAZ signaling axis has been found to be under the control of the differential expression of miR-21 [30]. Moreover, the increase of miR-21 levels appeared to be essential for down-regulation of YOD1 and the following destabilization of ITCH, which in turn augments the amount of LATS1/2 [30]. miRNA target prediction bioinformatics tools permit to recognize YAP1 also as a direct target of miR-21 (www.targetscan.org).

The relation between dystrophin and Hippo pathway could be more complex than thought, embracing not only the known structural mechanical role played by dystrophin, but also its signaling role. Dystrophin, as component of DGC, participates in transferring forces or loads produced in the muscle sarcomeres to extracellular matrix and integrates cell signaling in response to mechanical strain [36,37]. Moreover, YAP activity is regulated by mechanical signaling [38], YAP is a key-player in mechano-trasduction [4], and numerous YAP target genes encode proteins which link the cytoskeleton to sarcolemma and extracellular matrix [39].

**Fig 6:**
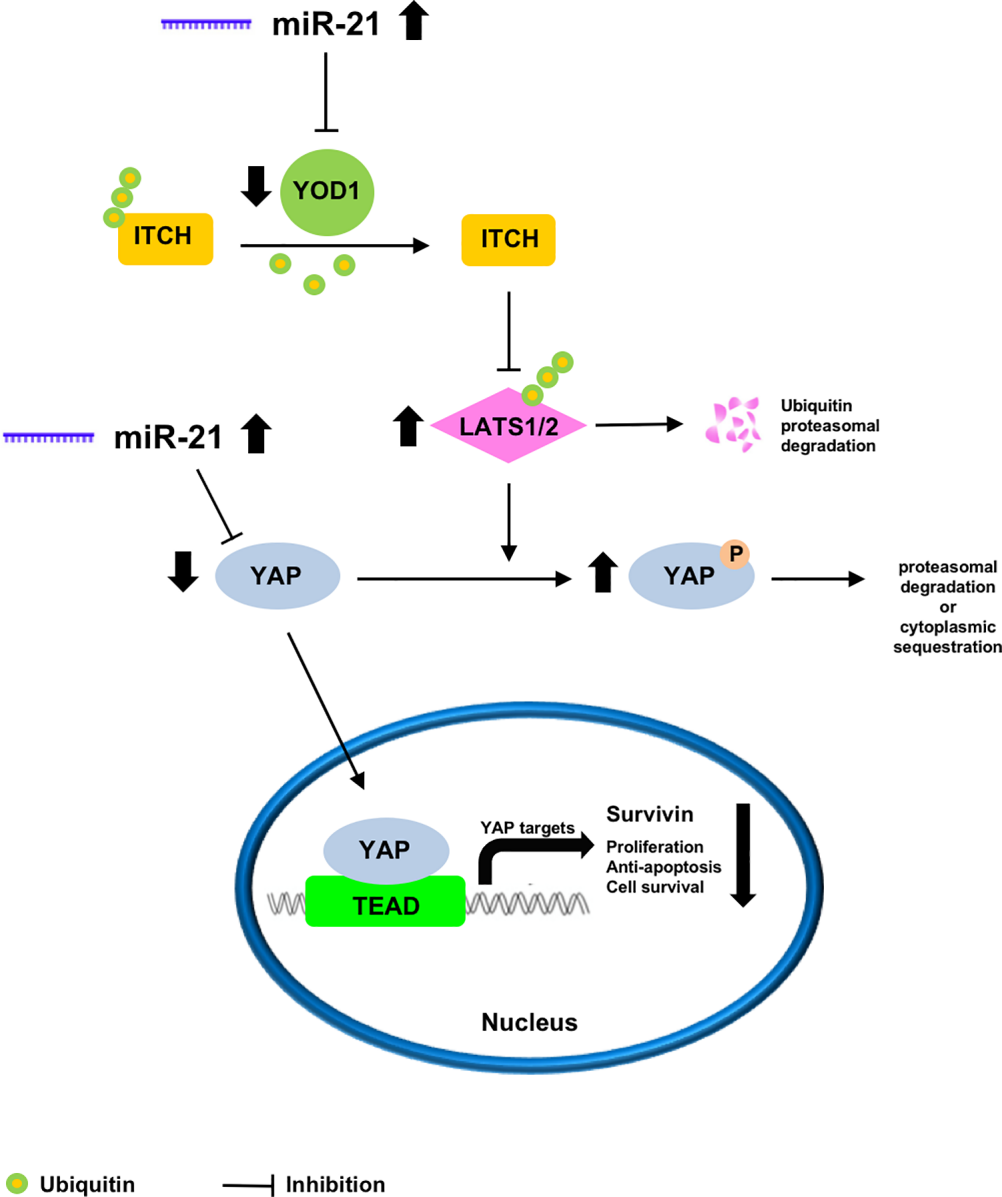
Proposed schematic model of Hippo pathway alterations in DMD. Low YOD1 resulting from increased inhibition by miR-21 leads to an increase of LATS1/2 activity which in turn augments phosphorylation of YAP. Reduced amount of active YAP, which is also a target of increased miR-21, causes decreased nuclear expression of YAP-mediated target genes.

Our novel results permit to postulate a schematic model of what might occur in the Hippo pathway in DMD muscles (Fig 6). Low levels of YOD1 resulting from increases in the level of miR-21 lead to an increased level of LATS1/2. Higher LATS1/2 activity enhances the potential for YAP phosphorylation, which reduces the amount of active YAP, which in turn is also a target of increased miR-21. The final consequence is that the nuclear expression of YAP-mediated target genes is reduced or shut down, with a negative result in proliferation, anti-apoptosis action and cell survival. Since YAP is involved in muscle development and regeneration [4], and regeneration features are progressively increased in DMD muscles until 6 years of age [40], our results are somewhat counterintuitive and increased activation of Hippo pathway should be expected. However, reduced YAP activity might be related to the known defective regenerative potential. In DMD necrotic changes are always found more numerous compared to regenerating fibres [36], and an inefficient regeneration is believed to rely on replicative senescence of satellite cells because of increased muscle fibre turnover and telomere shortening [41,42].

The proposed model suggests that several steps of the Hippo pathway may become therapeutic targets to treat DMD by using agonists or antagonists. Indeed, two faces of Hippo pathway have been identified for regenerative medicine and cancer treatment: whereas pharmacological inhibition of YAP activity might be a useful anticancer strategy, on the contrary YAP has beneficial roles in stimulating tissue repair and regeneration after injury so that its activation may be therapeutically beneficial [43]. Moreover, the Hippo pathway has been linked to various inflammatory modulators such as FoxO, TNFα, IL-6, COX2, AP-1, JAK and STAT, known to be involved in dystrophin-deficient pathogenic cascade, and both pre-clinical and clinical drugs of these signalling pathways have been recently reviewed [44]. The present study reports for the first time that Hippo signaling pathway is altered in DMD muscles, suggesting that some components could become novel therapeutic targets for DMD treatment. Further studies should investigate Hippo pathway in cultured dystrophic muscle cells and animal models after potential useful treatments.

## Supporting information

S1 Table S1 Dataset (humans). (DOCX)

S2 Table S2 Dataset (mice). (DOCX)
