# Expression of Concern: Hippo signaling pathway is altered in Duchenne muscular dystrophy

**DOI:** 10.1371/journal.pone.0306508

**Published:** 2024-06-27

**Authors:** The PLOS ONE Editors

After this article [1] was published, concerns were raised about Fig 1.

Specifically:

- Lanes 4–5 of the β-actin panel of Fig 1A appear similar to lanes 4–5 of the β-actin panel of Fig 1B when rotated 180 degrees.

Upon editorial follow-up, the original underlying western blot data for the article were not provided.

In light of the above concern, which cannot be resolved in the absence of the original underlying data, the *PLOS ONE* Editors issue this Expression of Concern.
