# Supplementary material for: Hippo signaling pathway is altered in Duchenne muscular dystrophy
Source: PLoS One. 2018 Oct 10;13(10):e0205514. doi: 10.1371/journal.pone.0205514 (PMC6179272; doi:10.1371/journal.pone.0205514)
Supplement: S1 Table — (DOCX) [file pone.0205514.s001.docx]

| **Subject number** | **Disease** | **Gender/Age (years)** | **YAP1** | **Phosphorylated YAP1** | **Survivin protein** | **Survivin mRNA** | **miR-21** |
| --- | --- | --- | --- | --- | --- | --- | --- |
| 1 | DMD | M/6 | 5213 | 14941 | 1950 | 1.2 | 12.7 |
| 2 | DMD | M/5 | 5098 | 15120 | 2054 | 0.92 | 12.054 |
| 3 | DMD | M/4 | 5132 | 15701 | 1916 | 1.12 | 12.514 |
| 4 | DMD | M/4 | 4982 | 15687 | 1828 | 0.86 | 12.320 |
| 5 | DMD | M/6 | 5017 | 12018 | 2090 | 1.001 | 13.020 |
| 6 | BMD | M/7 | 21125 | 8902 | 9905 | 2.3 | 6.026 |
| 7 | BMD | M/5 | 21314 | 7821 | 9300 | 2.1 | 5.759 |
| 8 | BMD | M/11 | 21450 | 8012 | 9528 | 2.6 | 6.229 |
| 9 | BMD | M/8 | 20987 | 8758 | 9690 | 2.3 | 5.727 |
| 10 | BMD | M/7 | 20859 | 8689 | 10094 | 2.8 | 5.122 |
| 11 | LGMD2A | M/14 | 21440 | 6651 | 13254 | 3.3 | 5.050 |
| 12 | LGMD2A | M/15 | 21981 | 6845 | 13128 | 2.8 | 5.087 |
| 13 | LGMD2A | F/24 | 21358 | 7154 | 12864 | 3.4 | 5.410 |
| 14 | LGMD2A | M/55 | 21874 | 7802 | 12892 | 3.2 | 4.938 |
| 15 | LGMD2A | F/52 | 22011 | 8108 | 12936 | 3.6 | 5.120 |
| 16 | LGMD2B | M/40 | 20154 | 7152 | 13981 | 2.9 | 5.080 |
| 17 | LGMD2B | F/50 | 21045 | 9810 | 13828 | 3.8 | 6.410 |
| 18 | LGMD2B | M/39 | 19987 | 6521 | 12981 | 3.95 | 5.938 |
| 19 | LGMD2B | M/44 | 20345 | 6892 | 12568 | 3.62 | 5.320 |
| 20 | LGMD2B | M/26 | 21089 | 7054 | 11987 | 4.1 | 4.930 |
| 21 | CTR | M/3 | 21440 | 6182 | 19241 | 3.3 | 2.530 |
| 22 | CTR | M/15 | 22080 | 4140 | 21986 | 3.2 | 3.785 |
| 23 | CTR | F/8 | 21606 | 8728 | 21015 | 2.95 | 4.800 |
| 24 | CTR | F/50 | 22008 | 8659 | 18987 | 3.87 | 4.049 |
| 25 | CTR | M/35 | 21792 | 11015 | 20892 | 3.72 | 3.360 |

**Legend**

DMD, Duchenne Muscular Dystrophy; BMD, Becker muscular dystrophy; LGMD2A, limb-girdle muscular dystrophy type 2A; LGMD2B, limb-girdle muscular dystrophy type 2B; CTR, control subjects
