# Supplementary material for: Hippo signaling pathway is altered in Duchenne muscular dystrophy
Source: PLoS One. 2018 Oct 10;13(10):e0205514. doi: 10.1371/journal.pone.0205514 (PMC6179272; doi:10.1371/journal.pone.0205514)
Supplement: S2 Table — (DOCX) [file pone.0205514.s002.docx]

| **Mouse** | **Type** | **Muscle** | **YAP1** | **Phosphorylated YAP1** |
| --- | --- | --- | --- | --- |
| 1 | wild-type | quadriceps | 6422 | 18871 |
| 2 | wild-type | quadriceps | 5854 | 18681 |
| 3 | wild-type | quadriceps | 6389 | 17748 |
| 4 | wild-type | quadriceps | 5986 | 18489 |
| 5 | mdx | quadriceps | 5312 | 28875 |
| 6 | mdx | quadriceps | 5756 | 28987 |
| 7 | mdx | quadriceps | 6004 | 29018 |
| 8 | mdx | quadriceps | 5286 | 29174 |
| 1 | wild-type | biceps | 21231 | 22257 |
| 2 | wild-type | biceps | 21125 | 22187 |
| 3 | wild-type | biceps | 21007 | 23989 |
| 4 | wild-type | biceps | 21081 | 22406 |
| 5 | mdx | biceps | 22365 | 28589 |
| 6 | mdx | biceps | 22428 | 28702 |
| 7 | mdx | biceps | 21989 | 27867 |
| 8 | mdx | biceps | 22182 | 28021 |
| 1 | wild-type | diaphragm | 19864 | 17865 |
| 2 | wild-type | diaphragm | 18983 | 17985 |
| 3 | wild-type | diaphragm | 19204 | 17543 |
| 4 | wild-type | diaphragm | 18724 | 18006 |
| 5 | mdx | diaphragm | 22876 | 21056 |
| 6 | mdx | diaphragm | 21985 | 21025 |
| 7 | mdx | diaphragm | 22147 | 22008 |
| 8 | mdx | diaphragm | 22058 | 22084 |
| 1 | wild-type | gastrocnemious | 18895 | 27892 |
| 2 | wild-type | gastrocnemious | 18759 | 27841 |
| 3 | wild-type | gastrocnemious | 19102 | 27659 |
| 4 | wild-type | gastrocnemious | 18754 | 28043 |
| 5 | mdx | gastrocnemious | 13854 | 28254 |
| 6 | mdx | gastrocnemious | 14187 | 29474 |
| 7 | mdx | gastrocnemious | 14168 | 29547 |
| 8 | mdx | gastrocnemious | 13235 | 29874 |
| 1 | wild-type | EDL | 17652 | 26006 |
| 2 | wild-type | EDL | 17875 | 25844 |
| 3 | wild-type | EDL | 16928 | 25841 |
| 4 | wild-type | EDL | 17208 | 25659 |
| 5 | mdx | EDL | 18424 | 29147 |
| 6 | mdx | EDL | 18224 | 29185 |
| 7 | mdx | EDL | 17186 | 29988 |
| 8 | mdx | EDL | 17981 | 29984 |
